# Molecular evidence for recent divergence of X- and Y-linked gene pairs in *Spinacia oleracea* L.

**DOI:** 10.1371/journal.pone.0214949

**Published:** 2019-04-09

**Authors:** Yosuke Okazaki, Satoshi Takahata, Hideki Hirakawa, Yutaka Suzuki, Yasuyuki Onodera

**Affiliations:** 1 The Graduate School of Agriculture, Hokkaido University, Sapporo, Japan; 2 Facility for Genome Informatics, Kazusa DNA Research Institute, Kazusa–kamatari, Kisarazu, Chiba, Japan; 3 The Department of Computational Biology and Medical Sciences, Graduate School of Frontier Sciences, The University of Tokyo, Kashiwa, Japan; 4 The Research Faculty of Agriculture, Hokkaido University, Sapporo, Japan; Chiba Daigaku, JAPAN

## Abstract

Dioecy has evolved recently and independently from cosexual populations in many angiosperm lineages, providing opportunities to understand the evolutionary process underlying this transition. Spinach (*Spinacia oleracea*) is a dioecious plant with homomorphic sex chromosomes (XY). Although most of the spinach Y chromosome recombines with the X chromosome, a region around the male-determining locus on Y does not recombine with its X counterpart, suggesting that this region might be related to the evolution of dioecy in the species. To identify genes located in the non-recombining region (MSY, male-specific region of Y), RNA-seq analysis of male and female progeny plants (eight each) from a sib-cross of a dioecious line was performed. We discovered only 354 sex-chromosomal SNPs in 219 transcript sequences (genes). We randomly selected 39 sex-chromosomal genes to examine the reproducibility of the RNA-seq results and observed tight linkage to the male-determining locus in a spinach segregating population (140 individuals). Further analysis using a large-scale population (>1400) and over 100 spinach germplasm accessions and cultivars showed that SNPs in at least 12 genes are fully linked to the male-determining locus, suggesting that the genes reside in the spinach MSY. Synonymous substitution rates of the MSY genes and X homologues predict a recent divergence (0.40 ± 0.08 Mya). Furthermore, synonymous divergence between spinach and its wild relative (*S*. *tetrandra*), whose sex chromosomes (XY) originated from a common ancestral chromosome, predicted that the species diverged around 5.7 Mya. Assuming that dioecy in *Spinacia* evolved before speciation within the genus and has a monophyletic origin, our data suggest that recombination around the spinach sex-determining locus might have stopped significantly later than the evolution of dioecy in *Spinacia*.

## Introduction

Cosexual systems (hermaphroditism and monoecy) are common in flowering plants, while dioecy is found in only a small percentage of angiosperms. However, dioecious species exhibit a wide taxonomic distribution and are often related to cosexual species, suggesting that cosexuality is the ancestral condition, from which dioecy has evolved recently and independently in many lineages. Sex determination in dioecious plants is often controlled genetically, and sex chromosomes have been found in 37 species belonging to 16 angiosperm families [1, 2]. The evolutionary transition from cosexuality to dioecy suggests that sex chromosomes originated from autosomes of ancestral cosexual species [1, 3].

Spinach (*Spinacia oleracea*) is usually considered dioecious, though certain cultivars, lines, genotypes, and crosses produce monoecious plants [4–6]. This vegetable is grown in over 50 countries (FAOSTAT, http://www.fao.org/faostat/en/#data/QC), and F1 hybrids are mainly used for spinach production in developed countries, e.g., Japan, the US, and countries in Europe [7]. Dioecism and monoecism are used in commercial hybrid seed production, and elucidation of the mechanisms underlying sex determination is an important issue for spinach breeding programs [8].

Self-fertilization of male spinach plants that occasionally produce seed generates progeny segregating into males and females, while selfing of female plants (highly female monoecious plants) does not produce male progeny, suggesting that males are the heterogametic sex (XY) and females are homogametic (XX) [9]. The sexual dimorphism in dioecious spinach lines has therefore been proposed to be controlled by an allelic pair, named *X* and *Y*. The male-determining locus (*Y*) has been mapped to the largest chromosome. The spinach sex chromosomes containing *X* and *Y*, i.e., X and Y, are homomorphic, recombining across most of their length, excluding the region around the *Y* locus [10–12].

Besides the cultivated species (spinach), *Spinacia* (2n = 12) includes two wild species, *S*. *turkestanica* and *S*. *tetrandra* [13]. Our molecular phylogenetic analysis showed that *S*. *oleracea* and *S*. *turkestanica* are closely related to each other; the latter may be a direct ancestor of the former [14]. *S*. *tetrandra* is phylogenetically distinct and clearly different in genome size and karyotype from the other *Spinacia* members. Furthermore, *S*. *tetrandra* has a heteromorphic sex chromosome pair (XY) and the other *Spinacia* members possess homomorphic sex chromosomes. The homomorphic and heteromorphic sex chromosomes are homologues, originating from a common ancestral chromosome [14]. In this context, it is reasonable to assume that dioecism of *Spinacia* members has a common origin and did not evolve independently. However, direct evidence is required to confirm this hypothesis.

Most members of the subfamily *Chenopodioideae*, a taxonomic group ranking above *Spinacia*, are cosexual, and the sister genus of *Spinacia*, *Blitum*, consists of gynomonoecious species [13, 15]. Furthermore, given that monoecy occurs in *S*. *oleracea*, dioecy in this genus likely arose via monoecy.

In evolutionary theory, the so-called two-mutation (two-factor) model [16–18] proposes that two mutations at closely linked loci are involved in the evolution of dioecy from cosexuality: one mutation (male sterility) yields females and the other (female sterility) suppresses female functions of the ancestral form, creating males. Suppression of recombination between the loci may be favored to maintain a stable fully sexually dimorphic population without cosexual or neuter individuals. Studies of *Silene*, *Asparagus*, and *Actinidia* spp. (kiwifruit) have supported the evolutionary predictions of the two-mutation model [19–24]. By contrast, a recent study suggested that sex determination in *Diospyros* species is controlled by a single gene; *OGI* small RNA from the Y chromosome functions as the male determinant, targeting the autosomal gene *MeGI*, which encodes a homeodomain transcription factor, and the *OGI–MeGI* interaction is assumed to regulate the development of female and male organs [25].

We recently showed that, in spinach, a Y-chromosomal region around the male-determining locus does not recombine with the counterpart region on the X chromosome [12, 26]. However, it is not clear whether the evolution of the non-recombining region on the spinach Y chromosome is directly associated with the establishment of dioecy in *Spinacia*, as predicted by the two-mutation (two-factor) model. Hereinafter, the non-recombining Y-chromosomal region flanked on both sides by pseudoautosomal regions (PARs), where X–Y crossover occurs frequently, is referred to as MSY (an abbreviation for the male-specific region of the Y chromosome), in accordance with a study of the human Y chromosome [27]. Furthermore, the part of the X chromosome without PARs is referred to as the X counterpart of MSY.

In this study, to estimate divergence time between the spinach MSY and its X counterpart by identifying X- and Y-linked gene pairs, progeny plants from sib-mating of a dioecious breeding line were subjected to RNA-seq and linkage analyses. Furthermore, to estimate the evolutionary age of dioecy in *Spinacia*, synonymous substitution rates between autosomal genes of *S*. *oleracea* and *S*. *tetrandra* were evaluated using RNA-seq reads obtained from the two species.

## Materials and methods

### Plant materials

Eight male and female spinach plants generated by a cross between individuals of the dioecious breeding line 03–009 (Tohoku Seed Co., Ltd., Utsunomiya, Tochigi, Japan) [6] were used for total RNA preparation and RNA-seq (S1 Table). The plants were grown in a growth chamber (LH-350S; Nippon Medical & Chemical Instruments Co. Ltd., Osaka, Japan) at 20°C under a 12 h photoperiod for 20 days (until 6–7 true leaves emerged) and were then grown under a 20 h photoperiod for the following 3 days.

For the linkage analysis, the plant populations (five populations, 1433 individuals in total) were the same as those used in a previous study (Table 1 in Kudoh et al. [26]), except for a backcross progeny population (BC_1_F_1_) from a cross between the breeding line 03–259 (Tohoku Seed Co. Ltd.) and the dioecious line 03–009, whose genomic DNA stock had run out. A total of 104 males, 102 females, and 6 monoecious plants were selected from unrelated material, including three spinach cultivars and 101 *Spinacia* germplasm accessions, and used to examine the association between the male phenotype and genes identified in this study. The plant materials from cultivars and germplasm accessions were used in our previous study (see S4 Table in Kudoh et al. [26]), except for a cultivar, Mazeran, whose DNA stock had run out.

### Total RNA preparation and RNA-seq analysis

Total RNA was extracted from whole aerial parts of each plant using an RNeasy Plant Mini Kit combined with an RNase-Free DNase Set (QIAGEN, Venlo, Netherlands). As shown in S1 Table, three female and three male RNA samples (Female 1–3 and Male 1–3) were prepared from single plants, and one female and one male RNA sample (Female 5 and Male 5) were prepared using equal amounts of RNA isolated from five individuals. Eight cDNA libraries were prepared from the RNA samples according to a method described in Takahata et al. [12] and sequenced using a HiSeq 2500 Instrument (Illumina, San Diego, CA, USA) configured to generate 100 bp paired-end reads.

### *De novo* transcriptome assembly, sex-chromosomal SNP identification, and expression analysis

After adapter trimming and quality filtering, RNA-seq reads from all eight RNA samples (S1 Table) were pooled and assembled using Trinity [28], which gave 249,622 cDNA contigs (S2 Table). Subsequently, non-redundant contigs (n = 190,200) were selected from the original contigs using RSEM 1.2.3. [29]. After the removal of sequence contamination (using the spinach chloroplast genome [AJ400848.1, NC_002202.1], *Arabidopsis thaliana* mitochondrial genome [NC_001284.2], *A*. *thaliana* RNAs from NCBI [741 rRNAs, 1690 tRNAs, 792 ncRNAs, 555 transcribed RNAs, 48 other RNAs, and 4 genomic RNAs], human genome [GRCh37], bacterial genomes [NCBI RefSeq], UniVec, fungal genomes [NCBI RefSeq], and PhiX174 [NC_001422.1]), a non-redundant unigene assembly (n = 189,948) was generated. The unigene assembly covered approximately 70% of protein-coding loci annotated in the spinach draft genome (spinach_CDS_v1.fa, ftp://www.spinachbase.org/pub/spinach) [30]. The clean reads for each RNA sample were separately mapped to the unigene assembly using Bowtie2 (2.2.2) [31]. The expression levels of unigenes in each sample were estimated by RPKM (reads per kb per million aligned reads). SNP calling and genotyping were performed using SAMtools (0.1.19) [32] and BCFtools (0.1.19) [33]. To avoid genotyping errors caused by a low read depth, when the reference (REF) depth was less than 10% of the total depth (reference [REF] + alternate [ALT]), the genotypes were judged to be homozygous for ALT alleles; in the opposite case, they were judged to be homozygous for REF alleles. The expression levels (log_2_RPKM) of each unigene in the female and male sample groups were compared using the ANOVA *F*-test. The expression ratio of Y- to X-linked alleles was estimated based on the total read depth of the four male samples. To identify nucleotide differences between *S*. *oleracea* and *S*. *tetrandra*, RNA-seq reads from *S*. *tetrandra* accession PI 647860 (SRR1766330) [34] were mapped to the spinach unigene assembly created in this study. Using WhatsHap (https://whatshap.readthedocs.io/en/latest/) [35], *S*. *tetrandra* allele sequences were generated based on the nucleotide variant information. To estimate nucleotide substitution rates and divergence time between *S*. *oleracea* and *S*. *tetrandra*, 100 unigenes were selected; these were associated with autosomal pseudomolecules (Chr. 1–3 and 5–6) in a spinach draft genome (spinach.genome.v1) [30] and were expressed at moderate levels in *S*. *tetrandra* (S3 Table).

### Estimation of nucleotide substitution rates and divergence time

Synonymous and non-synonymous substitution rates (*K*_S_ and *K*_A_) were calculated using MEGA7.0 [36] with the Nei and Gojobori method [37] and the Jukes–Cantor correction [38]. Divergence times, *T*, were estimated as *T* = *K*/2*r*, where *K* is the synonymous substitution rate (*K*_S_) and *r* is the neutral nucleotide substitution rate per site per generation (1 year per generation for spinach). To easily compare the XY divergence times of sex chromosomes between spinach and other plants, a neutral substitution rate of 6.5 × 10^−9^ per nucleotide per generation was used in accordance with a recent review article [3], which contains a list of the times of recombination suppression of sex-linked regions (XY divergence times) of various dioecious plants.

### DNA marker development and genetic map construction

Cleaved amplified polymorphic sequence (CAPS) and derived cleaved amplified polymorphic sequence (dCAPS) marker techniques [39] as well as an allele-specific amplification technique employing a 3′-terminal mismatch primer [40] were developed for SNP genotyping (S4 Table). A population (n = 140; ‘03-009-sib-cross B’ in Kudoh et al. [26]) generated by crosses between members of the dioecious line 03–009 was used for a linkage analysis of the DNA markers. A genetic map was constructed as described by Yamamoto et al. [41].

## Results and discussion

### Identification of sex-chromosomal SNPs and genes

RNA-seq reads from eight female and male progeny plants from the sib-mating were separately mapped to the spinach unigene assembly (reference transcriptome), with a mapping rate of 82.3% to 85.6%, a coverage of 50.4–65.0% of the reference, and an average read depth of 32× to 65× (S1 Table). As shown in Table 1 and S5 Table, sex-chromosomal SNPs were identified based on segregation patterns within the progeny, following the method described by Bergero and Charlesworth [42]. We identified 187 SNPs in 98 unigenes homozygous (X^a^X^a^) in all female progeny samples and heterozygous (X^a^Y^b^) in all male progeny samples (Segregation pattern 1 in Table 1 and S5 Table). Additionally, 167 SNPs in 125 unigenes were heterozygous (X^a^X^b^) in all of the female samples and homozygous (X^a^Y^a^) or hemizygous (X^a^Y^null^) in all of the male samples (Segregation pattern 2). Our analysis did not distinguish between X^a^Y^a^ and X^a^Y^null^, since the transcriptomes of the parental plants were not determined. No sex-chromosomal SNP with three alleles was found (Segregation pattern 3). Since four unigenes possessed SNPs that showed both Segregation patterns 1 and 2, the total number of the sex-chromosomal unigenes we identified was 219 (Table 1 and S5 Table).

**Table 1:** Identification of sex-chromosomal single nucleotide polymorphisms (SNPs).

Segregation pattern	Genotypes of parents		Genotypes of progeny		Numbers of SNPs	Numbers of unigenes
	Female	Male	Females	Males		
1	X^a^X^a^	X^a^Y^b^	X^a^X^a^	X^a^Y^b^	187	98
2	X^a^X^a^	X^b^Y^a^ or X^b^Y^null^	X^a^X^b^	X^a^Y^a^ or X^a^Y^null^	167	125
3	X^a^X^a^	X^b^Y^c^	X^a^X^b^	X^a^Y^c^	0	0
Total					354	219^†^

Eight females and males were employed to find sex-linked SNPs.

^†^Number does not include duplicates that carry both SNPs showing Segregation patterns 1 and 2.

BLASTX analysis against the NCBI NR database revealed that 191 of the 219 sex-chromosomal unigenes exhibited significant homology to known proteins and the remaining 28 showed no homology with any known polypeptide (S6 Table). Some of the 28 unigenes with no apparent coding function could represent long non-coding RNAs. However, further work is necessary to confirm this conclusion.

In the spinach draft genome assembly (spinach.genome.v1) [30], a pseudomolecule, chr4, most likely represents the sex chromosomes, since it shows extensive synteny with chromosomes 4 and 9 of *Beta vulgaris*, which were reported to have synteny with the spinach sex chromosomes in our previous study [12]. Based on a BLASTN homology search, contrary to our expectation, almost half (n = 115) of the sex-chromosomal unigenes were assigned not only to chr4, but also to other pseudomolecules (chr1–3 and 5–6). Among the pseudomolecules, chr4 had the most loci (n = 65) corresponding to the unigenes, followed by chr3 (n = 22) and chr2 (n = 11). The remaining half (n = 104) were located on contigs and scaffolds that are not incorporated into the pseudomolecules (Table 2 and S6 Table). Some unigenes, such as comp46713_c0_seq2 (chr3, e-value = 2.24E-84) and comp44081_c0_seq2 (chr1, e-value = 3.78E-82) (S6 Table), showed BLAST e-value scores, which may be insufficient to anchor them to the pseudomolecules. This might be due to the incompleteness of the spinach genome assembly (its total length without N spacers covers ~83% of the entire genome) and/or the inaccuracy of our transcriptome assembly. Further work is required to verify this result.

**Table 2:** Summary of the correspondence of 219 sex-chromosomal unigenes to pseudomolecules and scaffolds of the spinach draft genome.

Spinach_genome_v1	Number of unigenes
chr1	6
chr2	11
chr3	22
chr4	65
chr5	5
chr6	6
Scaffolds, contigs	104
Total	219

chr1–6, pseudomolecules.

**Fig 1:**
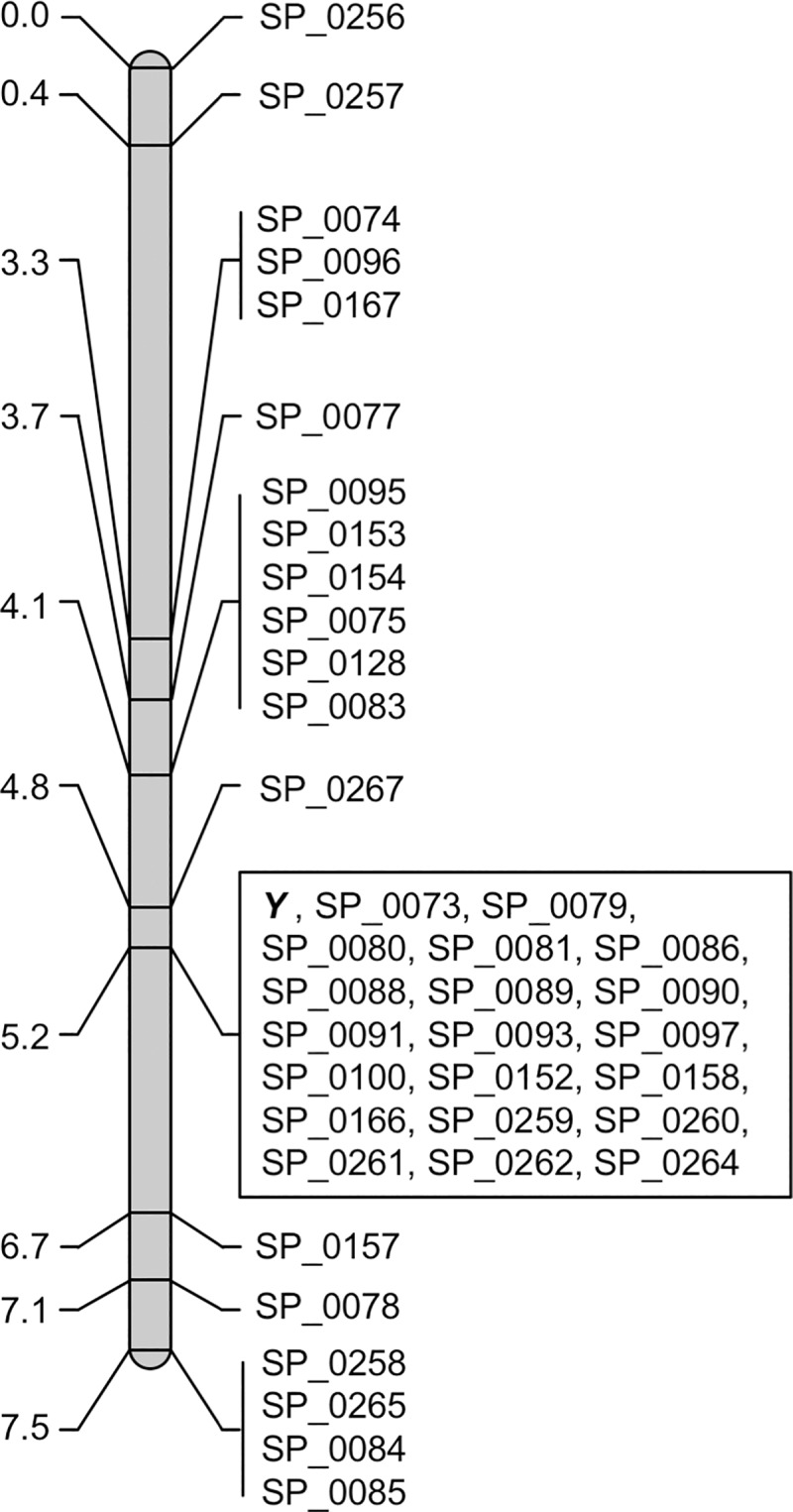
Genetic linkage map of SNP markers linked to the male-determining locus (*Y*). The map was constructed based on genotype data for the 03–009–sib–cross A population (68 males and 72 females) generated from sib-mating within a dioecious line, 03–009. Marker positions and names are shown to the left and right of the map.

To verify the validity of the results obtained by RNA-seq analysis of 16 individuals (eight males and eight females; S1 Table), we examined 39 randomly chosen unigenes from the 219 sex-chromosomal candidate genes using a sib-cross progeny population consisting of 140 individuals (03–009–sib–cross A; Table 3) in combination with SNP typing markers (CAPS, dCAPS, and allele-specific amplification markers; S4 Table). As shown in Fig 1, all of the unigenes were tightly linked to the male-determining locus in the mapping population (mapped to a 7.5 cM sex-chromosomal region). However, BLASTN analysis anchored 39 unigenes not only to the putative sex-chromosomal pseudomolecule (chr4) and scaffolds unincorporated into pseudomolecules, but also to the putative autosomal pseudomolecule (chr3) with significant e-values (Table 2, S4 and S6 Tables). This discrepancy might be due to inaccurate genome assembly. Further work is required to verify this result. However, the linkage analysis strongly supports the reliability and validity of the sex linkage inferred from our RNA-seq analysis. It is worth noting that 20 of the 39 unigenes co-segregated with the male-determining locus in the population. Therefore, subsequent analyses in this study were based on the results obtained by RNA-seq.

**Table 3:** Number of recombinants for the male-determining region and markers in the segregating populations.

Marker	Population				
	#1 (140)^†^	#2 (264)^†^	#3(473)^†^	#4 (415)^†^	#5 (141)^†^
SP_0073	0	0	0	0	0
SP_0079	0	0	0	0	0
SP_0080	0	0	0	0	0
SP_0081	0	0	0	0	0
SP_0086	0	0	0	0	0
SP_0088	0	0	0	0	0
SP_0089	0	0	n.v.	n.v.	n.v.
SP_0090	0	0	0	0	0
SP_0091	0	0	0	0	0
SP_0093	0	0	n.v.	n.v.	n.v.
SP_0097	0	0	0	0	0
SP_0100	0	0	0	0	0
SP_0152	0	0	n.v.	n.v.	n.v.
SP_0158	0	0	n.v.	n.v.	n.v.
SP_0166	0	0	0	0	0
SP_0259	0	0	0	0	0
SP_0260	0	0	0	0	0
SP_0261	0	0	0	0	0
SP_0262	0	0	n.v.	n.v.	n.v.
SP_0264	0	0	0	0	0

Numbers in parentheses indicate the population size.

n.v., no variant.

Population #1, 03–009–sib–cross A (68 males, 72 females).

Population #2, 03–009–sib–cross B (123 males, 141 females).

Population #3 03–259 × 03–009 BC_2_F_1_ (259 males, 214 females).

Population #4, 03–336 × 03–009 BC_1_F_1_ (209 males, 180 monoecious, 26 females).

Population #5 03–336 × 03–009 BC_2_F_1_ (70 males, 64 monoecious, 7 females).

^†^Kudoh et al. [26].

### Identification of genes located in the spinach MSY

To identify genes located in the spinach MSY and its X counterpart, linkage analysis of the 20 SNP markers (20 unigenes) that co-segregated with the male-determining locus was performed using four additional populations (1433 individuals in total, including 03–009–sib–cross A) (Table 3). We found variants at 15 of the co-segregating marker loci in all five populations, showing complete sex linkage. At the remaining five marker loci, variants were detected in only two populations, showing complete sex linkage in these populations, but no variant was found in the other three populations (Table 3).

Recombination frequencies vary among populations in several plants, including maize and Arabidopsis [43, 44]. In spinach, recombination frequencies around the sex-linked region vary between families from different cross combinations [41]. To screen out unigenes located in the regions (PARs) with ongoing recombination, the 20 SNP markers (unigenes) that co-segregated with the male phenotype (Table 3) were further examined for association with sex (the male-determining locus) using 104 males, 102 females, and 6 monoecious plants from three spinach cultivars and 101 *Spinacia* germplasm accessions (see Material and methods, and S4 Table in Kudoh et al. [26]).

As summarized in Table 4, 14 of the SNP markers were significantly associated with the male phenotype (Fisher’s exact test, *p* < 1E-10); however, the other six markers showed no or weak associations with the male phenotype (Fisher’s exact test, *p* > 1E-05). Among the 14 SNP markers (14 unigenes) with significant sex associations, 12 unigenes were strongly associated with sex (Fisher’s exact test, *p* < 1E-60) and were classified as MSY-located genes (Table 4). The eight SNP markers (eight unigenes) without significant sex associations (Fisher’s exact test, *p* > 1E-05) or with significant, but weak, associations with sex (Fisher’s exact test, *p* ≥ 6.4E-13) were considered to be located on a region with ongoing recombination or where recombination was very recently suppressed (Table 4). It is worth noting that the five SNP markers that showed no variation in the three populations in linkage analysis (Table 3) were not strongly associated with the male phenotype (Fisher’s exact test, *p* ≥ 6.4E-13) (Table 4). Taken together with the results of the linkage and association analyses (Fig 1 and Table 4), 27 unigenes (i.e., the 39 unigenes subjected to linkage analysis minus the 12 MSY genes) were classified as being located in PARs.

**Table 4:** Association between the male-determining locus and sex-chromosomal SNP markers, as determined by Fisher’s exact tests.

Marker	Unigene	Genotype						*p*-value
		Male			Female and monoecious			
		*aa*	*ab*	*bb*	*aa*	*ab*	*bb*	
SP_0073	comp18846_c0_seq1†	0	104	0	108	0	0	2.9E-63
SP_0079	comp32199_c0_seq1†	0	104	0	108	0	0	2.9E-63
SP_0080	comp32303_c0_seq1†	0	104	0	108	0	0	2.9E-63
SP_0081	comp32347_c0_seq1†	0	104	0	107	1	0	3.0E-61
SP_0086	comp41371_c0_seq2†	0	104	0	108	0	0	2.9E-63
SP_0088	comp45039_c0_seq1†	0	104	0	107	1	0	3.0E-61
SP_0089	comp45344_c0_seq1	102	2	0	108	0	0	0.24
SP_0090	comp47159_c0_seq1	0	104	0	11	97	0	8.0E-04
SP_0091	comp50243_c0_seq2†	0	104	0	108	0	0	2.9E-63
SP_0093	comp50644_c1_seq2	100	4	0	108	0	0	0.06
SP_0097	comp59512_c0_seq1	93	11	0	107	1	0	2.0E-03
SP_0100	comp50308_c0_seq1†	0	104	0	108	0	0	2.9E-63
SP_0152	comp33549_c0_seq1	68	34	2	107	0	1	6.4E-13
SP_0158	comp39641_c0_seq1	66	38	0	105	3	0	8.6E-11
SP_0166	comp49893_c0_seq1	6	98	0	4	104	0	0.53
SP_0259	comp41527_c0_seq1†	0	104	0	108	0	0	2.9E-63
SP_0260	comp49566_c0_seq4†	0	103	1	108	0	0	2.9E-63
SP_0261	comp43645_c0_seq1†	0	104	0	108	0	0	2.9E-63
SP_0262	comp49000_c0_seq6	103	1	0	108	0	0	0.49
SP_0264	comp34398_c0_seq1†	0	104	0	108	0	0	2.9E-63

^†^Genes classified as Y/X-linked genes located in the MSY and its X counterpart.

### Nucleotide substitution rates for pairs of X- and Y-linked alleles

**Fig 2:**
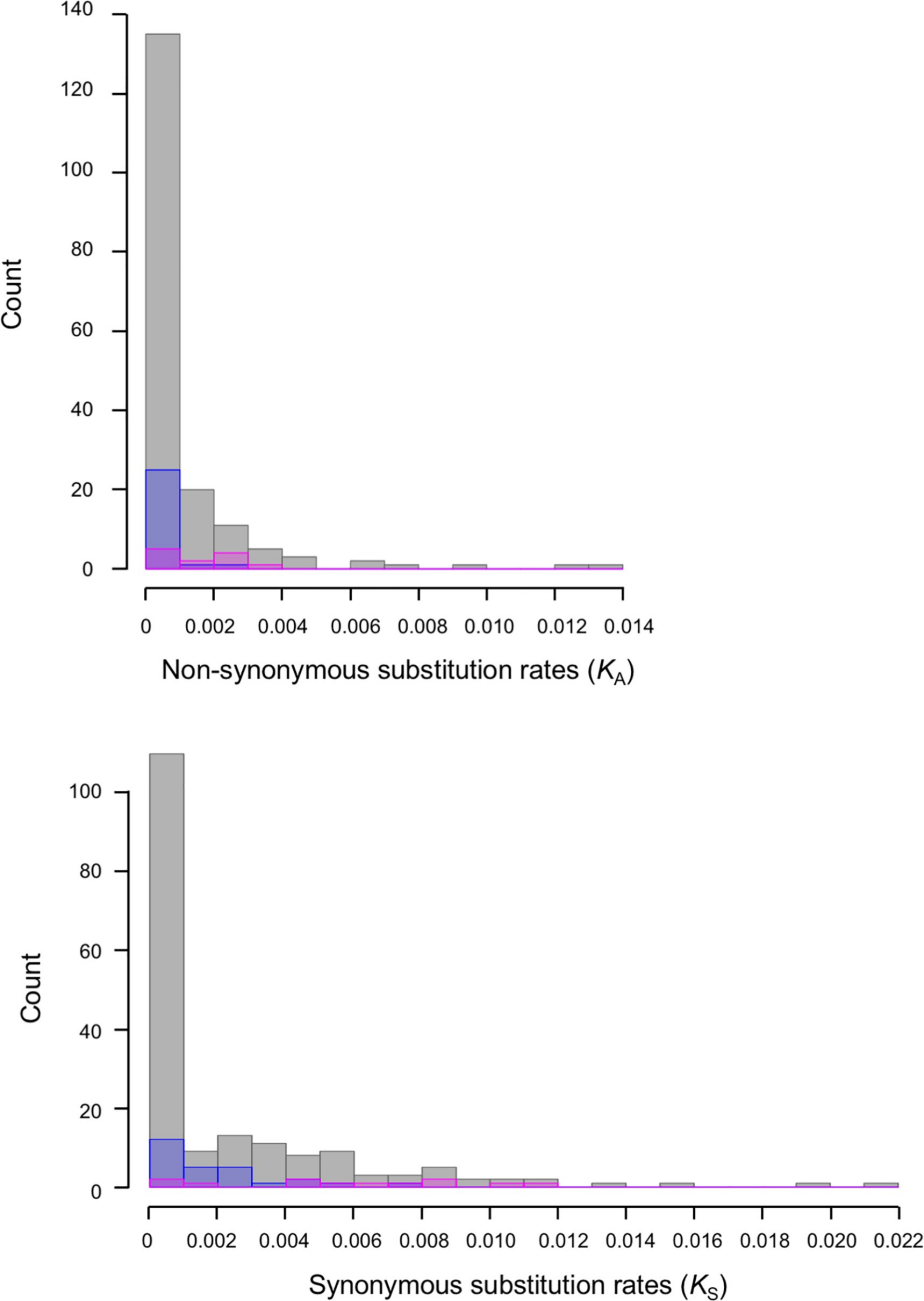
Distributions of synonymous and non-synonymous substitution rates for comparisons between allelic pairs of spinach sex-chromosomal genes. Gray, blue, and pink bars represent the 180 sex-chromosomal genes, 27 genes on the pseudoautosomal regions (PARs), and 12 X/Y-linked genes on the MSY and its X counterpart, respectively.

**Fig 3:**
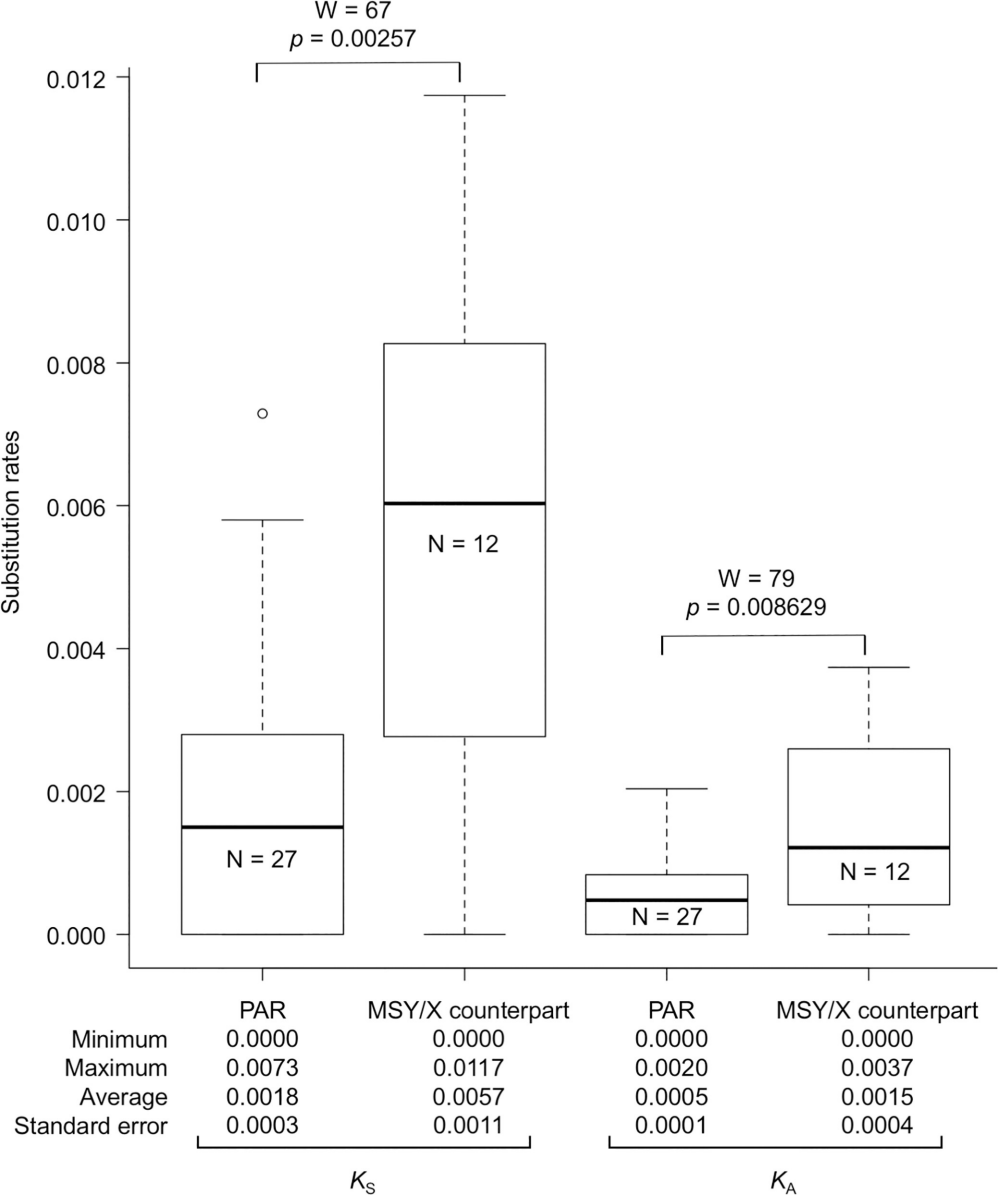
Synonymous and non-synonymous substitution rates for allelic pairs at loci on the MSY/X counterpart and the pseudoautosomal regions (PARs). Wilcoxon exact rank sum test results are given above the box plots.

To estimate the evolutionary time since the suppression of recombination at the male-determining locus, synonymous substitution rates (*K*_S_) were calculated for 180 of the sex-chromosomal allelic pairs in which ORFs were identified. As shown in Fig 2 and S7 Table, the values of *K*_S_ ranged from 0.000 to 0.0219. However, most of the values were approximately 0.0000 (the average *K*_S_ was 0.0022 [standard error: 0.0002]). Furthermore, the average *K*_S_ for the 12 X/Y-linked gene pairs located on the MSY and its X counterpart was 0.0057 (standard error: 0.0004), which was significantly higher than that (average *K*_S_ = 0.0018) of the 27 allelic pairs located in the PAR (Fig 3). Based on the *K*_S_ values for the 12 X/Y-linked gene pairs, the estimated divergence time between the MSY and its X-counterpart region was 0.40 ± 0.08 (average ± standard error) million years ago (Mya). The X/Y synonymous divergence rate (0.0057) in spinach is considerably lower than those in many other dioecious plants, including *Silene latifolia* (0.086), *Carica papaya* (0.024), and *Populus* (0.012), and is very close to that of sex-linked genes in the youngest evolutionary stratum (~0.4 Mya) formed since an X-autosome fusion in *Rumex hastatulus* [3, 45–47].

The average synonymous (*K*_S_) substitution rate for 100 autosomal genes for comparisons between *S*. *oleracea* and *S*. *tetrandra* was 0.0736 ± 0.0036 (average ± standard error), which corresponds to a divergence time of 5.66 ± 0.28 Mya (S3 Table). Given that dioecy of *Spinacia* evolved prior to the divergence of the two species, our results suggest that recombination stopped much later than the evolution of the male-determining factor(s). This notion does not contradict evolutionary theory predicting that, after the establishment of dioecy, sexually antagonistic mutation(s) around the sex-determining locus may favor suppressed recombination [3, 48]. The spinach sex chromosomes might have an evolutionary history similar to that of *Rumex hastatulus* sex chromosomes; in *Rumex*, suppressed recombination of the sex chromosomes is assumed to have evolved independently of dioecy [3, 45]. However, the possibility that the low XY divergence is due to very recent and independent evolution of dioecy and sex chromosomes in *S*. *oleracea* and *S*. *tetrandra* should also be considered because the evolutionary history of dioecy in *Spinacia* has not been fully elucidated.

A wide range of monoecism with various proportions of pistillate and staminate (or hermaphroditic) flowers exists in spinach [4, 6]. In certain monoecious spinach lines, the proportion of pistillate flowers per plant is considerably influenced by environmental conditions, particularly temperature; high temperatures result in a shift towards maleness, and vice versa [6, 49]. Furthermore, an increased dosage of the gene(s) responsible for monoecious expression results in a shift towards maleness [4, 6]. In this context, it is possible that the dioecism of *Spinacia* is regulated by a single-factor rather than a two-factor system, as in *Diospyros* [17, 25]. Assuming that extant *Spinacia* species originated from a monoecious ancestor with a sex-differentiation cascade by regulating the proportion of pistillate and staminate flowers, and that a single sex-determining factor upstream of the established cascade evolved in *Spinacia*, it seems reasonable that two mutations resulting in male and female sterilities, as predicted by the two-mutation model, are not necessarily needed for the evolution of dioecy in this genus [17, 50]. To reveal the evolutionary history of dioecy in *Spinacia*, a functional analysis of candidate genes for dioecism and monoecism (unpublished) is in progress.

Evolutionary theories predict that suppressed recombination reduces the efficacy of selection against deleterious mutations [48]. The theories suggest that Y-linked genes (on MSY) are likely to accumulate more deleterious mutations than X-linked genes (on the X counterpart of MSY) and genes located on recombining autosomal and pseudoautosomal regions. However, although the average non-synonymous substitution rate (*K*_A_) for the 12 X/Y-linked gene pairs was significantly higher than that for the 27 allelic pairs located in the PAR (Fig 3), there were no significant differences in the *K*_A_/*K*_S_ values of the X/Y-linked gene pairs (0.274) and the pseudoautosomal allelic pairs (0.209) (*W* = 69, *p* = 0.43). Nevertheless, to better answer this question, further investigation using homologous outgroup sequences will be necessary.

To verify whether mutations resulting in reduced expression occurred in Y-linked genes, the ratio of Y/X expression of sex-linked genes with multiple SNPs (n > 1) heterozygous in all male plants used for the RNA-seq analysis was estimated. As shown in S8 Table, the Y- and X-linked genes located on the MSY and its X-counterpart region were expressed at approximately the same level (on average, Y/X = 0.99, standard error: 0.04) in male plants. Furthermore, the average expression ratio of the Y/X-linked genes was not significantly different from that (0.98) of the allelic pairs for the PAR genes or from that (0.98) of the sex-chromosomal allelic pairs (S8 Table). Thus, there was no obvious evidence for the deterioration of Y-linked genes. Our data suggest that the tempo of Y deterioration could differ among taxa, although further investigations will be required to examine this notion. In *Rumex hastatulus*, the expression levels of Y-linked genes are lower than those of X-linked genes, located in the young evolutionary stratum (~0.4 Mya) [3, 45].

Consistent with the above results, for the 12 X/Y-linked genes other than comp45039_c0_seq1, there were no significant differences in expression levels between sexes (S1 Fig). comp45039_c0_seq1 (encoding xyloglucan endotransglucosylase/hydrolase protein 9) exhibited slightly higher (1.3-fold) expression in males than in females (*F* = 18.07, *df* = 6, *p* = 0.005), though the ratio of Y/X expression in males was 1.01. Our transcriptome data showed that 6.8% (n = 2349) of the unigene assembly (34411 unigenes [>400 nt]) were significantly differentially expressed between sexes at *p* < 0.01, a fraction of which might be directly or indirectly influenced by the sex-determining locus. The sex-chromosomal genes also include genes with sex-biased expression (9.2%). comp45039_c0_seq1 might be one such gene with sex-biased expression.

Recent studies suggested that the Y chromosome lost many functional genes present in the X chromosome in *Silene latifolia* and *Rumex* species. Although certain subsets of their sex-linked hemizygous genes show nearly complete dosage compensation, other subsets are not, or only partially, dosage-compensated [51, 52]. Using our approach, we were not able to comprehensively identify sex-linked genes hemizygous in males (only an X allele in male); accordingly, it is unclear whether gene loss from Y chromosomes and/or the transposition of genes from autosomes to the X chromosome occurred during the evolution of spinach. Given that hemizygous genes are dosage-compensated locally rather than globally as is the case in *Silene latifolia* and *Rumex* species [51, 52], the expression levels of a certain proportion of these genes are expected to be lower in males than in females. However, among the 125 unigenes showing Segregation pattern 2 (Table 1), we found only two genes (comp45718_c0_seq1 and comp50876_c1_seq3) with significant female-biased expression (*p* < 0.01, ANOVA); the male/female ratios of comp45718_c0_seq1 and comp50876_c1_seq3 were 0.62 (*F* = 27.692, *df* = 6, *p* = 0.0019) and 0.50 (*F* = 72.896, *df* = 6, *p* = 0.0001), respectively (S2 Fig). Based on these transcriptome data and the short history of the spinach MSY inferred from the low XY divergence, it is reasonable to assume that only a small portion of sex-linked genes are hemizygous in males. However, as pointed out by Papadopulos et al. [51], further analyses using both transcriptome- and genome-based approaches are required to precisely estimate the extent of gene loss from the spinach Y chromosome.

## Supporting information

S1 Fig Comparison between the expression levels of the 12 X/Y-linked genes in male and female plants. Broken lines represent 2-fold differences. Closed circles indicate significant differences at *p* < 0.01.
(PDF)

S2 Fig Comparison of the expression levels of sex-chromosomal genes between male and female plants. Plots represent expression levels of the 125 sex-chromosomal genes showing Segregation pattern 2 (see Table 1). Broken lines represent 2-fold differences. Closed red and black circles represent genes with significant female- and male-biased expression at *p* < 0.01.
(PDF)

S1 Table Summary of RNA samples and RNA-seq reads used to identify sex-linked genes. (DOCX)

S2 Table Summary statistics for the *de novo* transcriptome assembly of Illumina reads from the dioecious line 03–009 obtained using Trinity. (DOCX)

S3 Table Nucleotide substitution rates for the comparison between *S*. *oleracea* and *S*. *tetrandra* genes. (XLSX)

S4 Table SNP typing markers developed in this study. (XLSX)

S5 Table Sex-chromosomal unigenes identified by an RNA-seq analysis of eight females and males from the dioecious line 03–009. (DOCX)

S6 Table BLAST search for spinach sex-chromosomal unigenes. (XLSX)

S7 Table Nucleotide substitution rates for alternative alleles at the sex-chromosomal loci. (XLSX)

S8 Table Expression ratio between alternative alleles at sex-chromosomal loci in male plants. (DOCX)
