# Supplementary figures and images for: Molecular evidence for recent divergence of X- and Y-linked gene pairs in Spinacia oleracea L
Source: PLoS One. 2019 Apr 9;14(4):e0214949. doi: 10.1371/journal.pone.0214949 (PMC6456208; doi:10.1371/journal.pone.0214949)

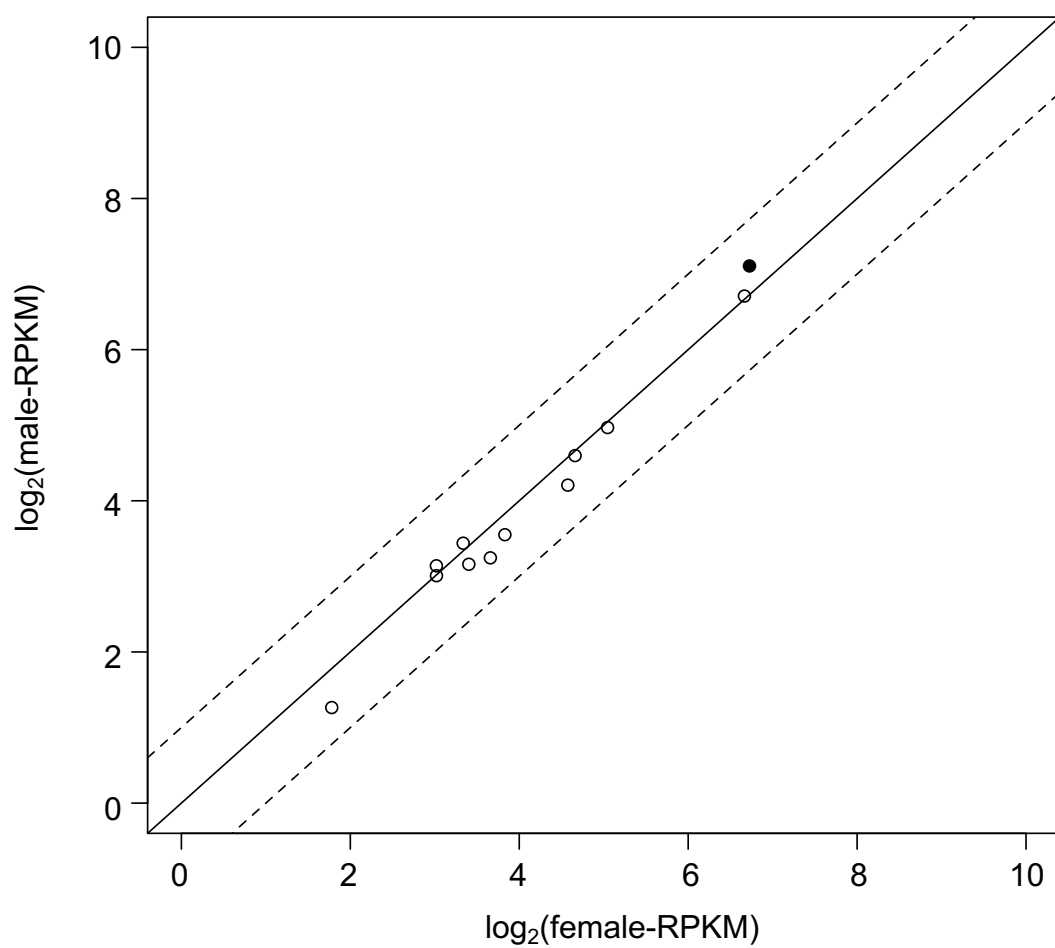

S1 Fig

Supplement: S1 Fig — Broken lines represent 2-fold differences. Closed circles indicate significant differences at p < 0.01. (PDF) [file pone.0214949.s001.pdf]

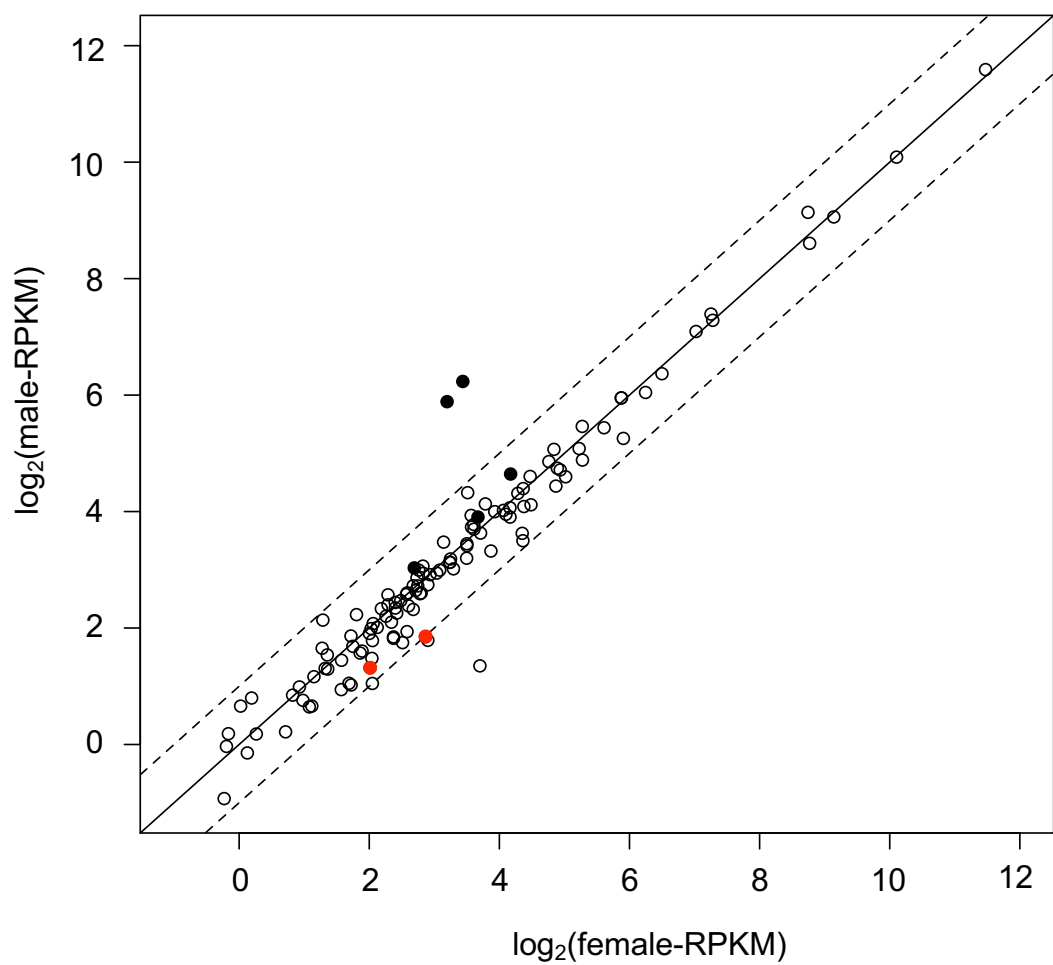

S2 Fig

Supplement: S2 Fig — Plots represent expression levels of the 125 sex-chromosomal genes showing Segregation pattern 2 (see Table 1). Broken lines represent 2-fold differences. Closed red and black circles represent genes with significant female- and male-biased expression at p < 0.01. (PDF) [file pone.0214949.s002.pdf]
