# Supplementary material for: Molecular evidence for recent divergence of X- and Y-linked gene pairs in Spinacia oleracea L
Source: PLoS One. 2019 Apr 9;14(4):e0214949. doi: 10.1371/journal.pone.0214949 (PMC6456208; doi:10.1371/journal.pone.0214949)
Supplement: S1 Table — (DOCX) [file pone.0214949.s003.docx]

**S1 Table. RNA samples and RNA-seq reads used to identify sex-linked genes.**

| Sample name^†^ | Number of plants | SRA ID | Input reads | Mapped reads | Mapping rate (%) | Coverage against reference transcriptome (%) | Mean coverage depth (fold) |
| --- | --- | --- | --- | --- | --- | --- | --- |
| Female 1 | 1 | DRA002974 | 42,935,273 | 36,672,403 | 85.41 | 63.80 | 54.88 |
| Female 2 | 1 | DRA002975 | 31,295,399 | 26,498,412 | 84.67 | 61.03 | 39.66 |
| Female 3 | 1 | DRA002976 | 51,074,955 | 43,548,112 | 85.26 | 65.03 | 65.18 |
| Female 5 | 5 | DRA002977 | 43,729,009 | 37,187,437 | 85.04 | 64.62 | 55.66 |
| Male 1 | 1 | DRA002978 | 25,192,859 | 21,567,686 | 85.61 | 56.26 | 32.28 |
| Male 2 | 1 | DRA002979 | 46,103,784 | 37,939,873 | 82.29 | 50.44 | 56.78 |
| Male 3 | 1 | DRA002980 | 27,479,268 | 23,377,250 | 85.07 | 57.91 | 34.99 |
| Male 5 | 5 | DRA002981, DRA004582 | 27,752,167 | 23,761,814 | 85.62 | 57.23 | 35.56 |

^†^The total RNA samples were prepared from female and male plants in a sib-cross progeny of dioecious line 03-009.
