# Supplementary material for: Molecular evidence for recent divergence of X- and Y-linked gene pairs in Spinacia oleracea L
Source: PLoS One. 2019 Apr 9;14(4):e0214949. doi: 10.1371/journal.pone.0214949 (PMC6456208; doi:10.1371/journal.pone.0214949)
Supplement: S2 Table — (DOCX) [file pone.0214949.s004.docx]

**S2 Table. Summary statistics for the *de novo* transcriptome assembly of Illumina reads from the dioecious line 03-009 obtained using Trinity.**

|  | Original contigs | Non-redundant contigs^a^ | Non-redundant unigenes^b^ |
| --- | --- | --- | --- |
| Number of contigs | 249,622 | 190,200 | 189,948 |
| Total length of contigs | 133,464,632 | 66,816,943 | 66,030,647 |
| Average length of contigs | 534 | 351 | 347 |
| Maximum length of contigs | 34,519 | 34,073 | 15,770 |
| Minimum length of contigs | 101 | 101 | 101 |
| N50 length | 1,474 | 607 | 591 |

^a^Non-redundant contigs were selected from the original contigs using RSEM 1.2.3.

^b^Non-redundant unigenes were generated by removal of contaminating sequences (using the spinach chloroplast genome [AJ400848.1, NC_002202.1], *A. thaliana* mitochondrial genome [NC_001284.2], *A. thaliana* RNAs from NCBI [741 rRNAs, 1690 tRNAs, 792 ncRNAs, 555 transcribed RNAs, 48 other RNAs, and 4 genomic RNAs], human genome [GRCh37], bacterial genomes [NCBI RefSeq], UniVec, fungal genomes [NCBI RefSeq], and PhiX174 [NC_001422.1]) from the non-redundant contigs.
