# Supplementary material for: Molecular evidence for recent divergence of X- and Y-linked gene pairs in Spinacia oleracea L
Source: PLoS One. 2019 Apr 9;14(4):e0214949. doi: 10.1371/journal.pone.0214949 (PMC6456208; doi:10.1371/journal.pone.0214949)
Supplement: S5 Table — (DOCX) [file pone.0214949.s007.docx]

**S5 Table. Sex-chromosomal unigenes identified by an RNA-seq analysis of eight females and males from the dioecious line 03-009.**

| Unigene | Length (bp) | Number of SNPs | SNP position | Genotype | | Segregation pattern^†^ |
| --- | --- | --- | --- | --- | --- | --- |
|  |  |  |  | Female | Male |  |
| comp18640_c0_seq1 | 944 | 1 | 62 | GG | GA | 1 |
| comp18846_c0_seq1 | 1,729 | 1 | 1,021 | AA | AG | 1 |
| comp18855_c0_seq1 | 2,047 | 3 | 470 | CC | CT | 1 |
|  |  |  | 791 | TT | TC | 1 |
|  |  |  | 1,557 | GG | GA | 1 |
| comp19087_c1_seq1 | 997 | 1 | 830 | AA | AG | 1 |
| comp21013_c0_seq1 | 1,821 | 1 | 252 | TT | TC | 1 |
| comp21424_c0_seq1 | 1,082 | 2 | 844 | TT | TC | 1 |
|  |  |  | 917 | AA | AC | 1 |
| comp22234_c0_seq1 | 3,118 | 1 | 1,489 | TT | TC | 1 |
| comp23335_c0_seq1 | 847 | 1 | 381 | GG | GT | 1 |
| comp26781_c0_seq1 | 1,580 | 1 | 313 | GG | GT | 1 |
| comp29361_c0_seq1 | 158 | 1 | 107 | CC | CT | 1 |
| comp32199_c0_seq1 | 1,343 | 1 | 719 | AA | AG | 1 |
| comp32303_c0_seq1 | 1,192 | 4 | 201 | TT | TC | 1 |
|  |  |  | 405 | CC | CT | 1 |
|  |  |  | 439 | TT | TC | 1 |
|  |  |  | 952 | TT | TA | 1 |
| comp32347_c0_seq1 | 1,518 | 2 | 856 | TT | TC | 1 |
|  |  |  | 961 | TT | TG | 1 |
| comp32433_c0_seq1 | 1,711 | 1 | 1509 | GG | GC | 1 |
| comp32454_c0_seq1 | 2,140 | 2 | 856 | TT | TA | 1 |
|  |  |  | 1448 | GG | GA | 1 |
| comp33549_c0_seq1 | 1,925 | 2 | 521 | CC | CA | 1 |
|  |  |  | 1,256 | TT | TA | 1 |
| comp33576_c0_seq1 | 389 | 1 | 202 | GG | GA | 1 |
| comp33723_c0_seq1 | 3,006 | 2 | 371 | GG | GC | 1 |
|  |  |  | 1,728 | GG | GA | 1 |
| comp33872_c0_seq1 | 379 | 1 | 123 | AA | AC | 1 |
| comp34398_c0_seq1 | 2,121 | 3 | 728 | GG | GA | 1 |
|  |  |  | 729 | GG | GA | 1 |
|  |  |  | 1,895 | GG | GA | 1 |
| comp36480_c1_seq2 | 1,546 | 1 | 1,267 | GG | GA | 1 |
| comp37879_c0_seq1 | 7,154 | 1 | 1,405 | TT | TC | 1 |
| comp38376_c0_seq1 | 3,298 | 2 | 2,760 | TT | TC | 1 |
|  |  |  | 2,923 | GG | GA | 1 |
| comp38649_c0_seq1 | 1,098 | 2 | 660 | AA | AT | 1 |
|  |  |  | 920 | GG | GA | 1 |
| comp39327_c0_seq1 | 2,434 | 1 | 2,167 | TT | TG | 1 |
| comp39602_c0_seq2 | 1,684 | 2 | 109 | TT | TA | 1 |
|  |  |  | 110 | CC | CT | 1 |
| comp39641_c0_seq1 | 5,552 | 2 | 485 | AA | AT | 1 |
|  |  |  | 3,777 | GG | GA | 1 |
| comp39876_c0_seq2 | 2,284 | 1 | 2,056 | GG | GA | 1 |
| comp40573_c0_seq1 | 1,944 | 1 | 1,335 | TT | TC | 1 |
| comp41154_c0_seq1 | 719 | 1 | 264 | AA | AC | 1 |
| comp41371_c0_seq2 | 522 | 1 | 110 | AA | AG | 1 |
| comp41476_c0_seq1 | 1,141 | 2 | 321 | CC | CT | 1 |
|  |  |  | 938 | AA | AG | 1 |
| comp41527_c0_seq1 | 1,529 | 3 | 756 | TT | TG | 1 |
|  |  |  | 822 | TT | TA | 1 |
|  |  |  | 1,066 | CC | CT | 1 |
| comp42108_c0_seq1 | 422 | 2 | 273 | CC | CT | 1 |
|  |  |  | 338 | AA | AG | 1 |
| comp43645_c0_seq1 | 1,638 | 3 | 742 | GG | GA | 1 |
|  |  |  | 1,307 | CC | CT | 1 |
|  |  |  | 1,503 | AA | AC | 1 |
| comp44743_c0_seq1 | 3,737 | 1 | 3,391 | GG | GA | 1 |
| comp44781_c0_seq3 | 2,189 | 2 | 1,593 | AA | AG | 1 |
|  |  |  | 2,006 | GG | GT | 1 |
| comp45039_c0_seq1 | 1,821 | 5 | 482 | CC | CG | 1 |
|  |  |  | 648 | AA | AT | 1 |
|  |  |  | 1,013 | CC | CT | 1 |
|  |  |  | 1,186 | AA | AG | 1 |
|  |  |  | 1,255 | AA | AC | 1 |
| comp45344_c0_seq1 | 7,957 | 3 | 2,756 | TT | TA | 1 |
|  |  |  | 3,144 | TT | TC | 1 |
|  |  |  | 5,852 | AA | AT | 1 |
| comp45700_c0_seq2 | 327 | 2 | 97 | AA | AG | 1 |
|  |  |  | 173 | AA | AC | 1 |
| comp46724_c0_seq1 | 2,644 | 1 | 1,280 | TT | TA | 1 |
| comp46844_c0_seq1 | 956 | 1 | 274 | CC | CA | 1 |
| comp46846_c0_seq2 | 367 | 1 | 225 | AA | AG | 1 |
| comp47159_c0_seq1 | 3,795 | 3 | 501 | AA | AT | 1 |
|  |  |  | 572 | GG | GT | 1 |
|  |  |  | 2,316 | TT | TG | 1 |
| comp47416_c0_seq2 | 314 | 2 | 90 | AA | AG | 1 |
|  |  |  | 166 | AA | AC | 1 |
| comp47931_c0_seq4 | 1,120 | 1 | 789 | AA | AG | 1 |
| comp47939_c1_seq1 | 1,569 | 1 | 706 | GG | GA | 1 |
| comp48410_c1_seq1 | 555 | 1 | 198 | AA | AG | 1 |
| comp49000_c0_seq6 | 4,001 | 2 | 647 | TT | TC | 1 |
|  |  |  | 2,610 | AA | AT | 1 |
| comp49436_c0_seq1 | 3,049 | 1 | 2,193 | AA | AG | 1 |
| comp49566_c0_seq4 | 3,488 | 3 | 526 | GG | GT | 1 |
|  |  |  | 2,497 | TT | TC | 1 |
|  |  |  | 3,054 | TT | TG | 1 |
| comp49789_c0_seq1 | 1,088 | 1 | 478 | GG | GT | 1 |
| comp49795_c0_seq1 | 1,098 | 1 | 502 | TT | TG | 1 |
| comp49893_c0_seq1 | 2,003 | 1 | 1,668 | CC | CA | 1 |
| comp49974_c0_seq2 | 1,698 | 4 | 751 | AA | AC | 1 |
|  |  |  | 753 | TT | TA | 1 |
|  |  |  | 769 | TT | TG | 1 |
|  |  |  | 783 | CC | CT | 1 |
| comp50197_c0_seq1 | 1,104 | 1 | 647 | TT | TC | 1 |
| comp50243_c0_seq2 | 1,744 | 4 | 894 | GG | GA | 1 |
|  |  |  | 1,035 | GG | GT | 1 |
|  |  |  | 1,157 | TT | TC | 1 |
|  |  |  | 1,325 | CC | CT | 1 |
| comp50308_c0_seq1 | 2,874 | 8 | 1,058 | GG | GC | 1 |
|  |  |  | 1,191 | GG | GA | 1 |
|  |  |  | 1,397 | TT | TA | 1 |
|  |  |  | 1,485 | AA | AC | 1 |
|  |  |  | 1,565 | TT | TC | 1 |
|  |  |  | 1,646 | GG | GT | 1 |
|  |  |  | 1,866 | AA | AG | 1 |
|  |  |  | 1,982 | AA | AC | 1 |
| comp50644_c1_seq2 | 957 | 1 | 415 | CC | CA | 1 |
| comp50659_c0_seq3 | 2,710 | 2 | 1,758 | CC | CA | 1 |
|  |  |  | 2,470 | AA | AG | 1 |
| comp50662_c0_seq3 | 3,622 | 1 | 1,546 | TT | TG | 1 |
| comp50740_c0_seq1 | 1,558 | 3 | 565 | TT | TC | 1 |
|  |  |  | 604 | AA | AC | 1 |
|  |  |  | 661 | AA | AC | 1 |
| comp50773_c0_seq3 | 1,366 | 1 | 1,020 | GG | GA | 1 |
| comp50843_c0_seq2 | 1,675 | 2 | 478 | AA | AG | 1 |
|  |  |  | 540 | GG | GC | 1 |
| comp50844_c0_seq1 | 3,662 | 1 | 2,620 | AA | AC | 1 |
| comp50907_c0_seq4 | 4,176 | 9 | 251 | TT | TC | 1 |
|  |  |  | 770 | TT | TA | 1 |
|  |  |  | 820 | TT | TC | 1 |
|  |  |  | 1,722 | GG | GC | 1 |
|  |  |  | 1,747 | TT | TG | 1 |
|  |  |  | 2,310 | CC | CA | 1 |
|  |  |  | 2,732 | TT | TA | 1 |
|  |  |  | 3,126 | TT | TA | 1 |
|  |  |  | 3,454 | GG | GC | 1 |
| comp50953_c0_seq8 | 2,764 | 2 | 1,289 | AA | AG | 1 |
|  |  |  | 1,652 | AA | AG | 1 |
| comp51118_c0_seq3 | 649 | 3 | 375 | GG | GA | 1 |
|  |  |  | 467 | TT | TC | 1 |
|  |  |  | 516 | GG | GA | 1 |
| comp51420_c0_seq2 | 1,318 | 5 | 166 | GG | GC | 1 |
|  |  |  | 275 | AA | AC | 1 |
|  |  |  | 992 | CC | CT | 1 |
|  |  |  | 1,040 | GG | GT | 1 |
|  |  |  | 1,041 | GG | GT | 1 |
| comp51453_c0_seq1 | 3,292 | 2 | 2,788 | TT | TC | 1 |
|  |  |  | 2,857 | AA | AC | 1 |
| comp51683_c8_seq16 | 1,738 | 1 | 72 | AA | AC | 1 |
| comp51767_c0_seq2 | 1,898 | 1 | 1,643 | AA | AG | 1 |
| comp51849_c4_seq1 | 2,073 | 1 | 1,780 | AA | AT | 1 |
| comp51901_c1_seq31 | 1,231 | 1 | 167 | TT | TG | 1 |
| comp51959_c0_seq3 | 2,058 | 1 | 1,878 | GG | GT | 1 |
| comp52055_c0_seq1 | 2,635 | 4 | 2,431 | AA | AG | 1 |
|  |  |  | 2,432 | TT | TC | 1 |
|  |  |  | 2,433 | GG | GA | 1 |
|  |  |  | 2,447 | AA | AC | 1 |
| comp52201_c0_seq3 | 3,905 | 2 | 330 | CC | CT | 1 |
|  |  |  | 339 | TT | TG | 1 |
| comp52213_c0_seq1 | 1,074 | 1 | 846 | AA | AG | 1 |
| comp52326_c1_seq8 | 1,764 | 1 | 302 | GG | GC | 1 |
| comp52369_c1_seq1 | 439 | 1 | 194 | TT | TA | 1 |
| comp52408_c0_seq4 | 3,205 | 1 | 2,365 | TT | TC | 1 |
| comp52474_c2_seq2 | 4,430 | 8 | 581 | AA | AC | 1 |
|  |  |  | 602 | CC | CA | 1 |
|  |  |  | 645 | GG | GA | 1 |
|  |  |  | 1,248 | GG | GT | 1 |
|  |  |  | 1,449 | GG | GA | 1 |
|  |  |  | 3,348 | GG | GA | 1 |
|  |  |  | 3,363 | CC | CA | 1 |
|  |  |  | 3,374 | GG | GA | 1 |
| comp52503_c0_seq1 | 1,301 | 4 | 787 | CC | CT | 1 |
|  |  |  | 791 | CC | CT | 1 |
|  |  |  | 858 | CC | CG | 1 |
|  |  |  | 884 | AA | AG | 1 |
| comp52531_c4_seq14 | 516 | 2 | 58 | AA | AC | 1 |
|  |  |  | 60 | GG | GC | 1 |
| comp52540_c0_seq9 | 474 | 1 | 103 | AA | AG | 1 |
| comp52545_c0_seq18 | 2,417 | 4 | 201 | GG | GA | 1 |
|  |  |  | 688 | AA | AG | 1 |
|  |  |  | 963 | CC | CT | 1 |
|  |  |  | 1,005 | AA | AG | 1 |
| comp52577_c1_seq14 | 3,363 | 2 | 3,164 | AA | AT | 1 |
|  |  |  | 3,233 | TT | TC | 1 |
| comp52579_c1_seq1 | 1,046 | 1 | 241 | GG | GA | 1 |
| comp52587_c1_seq71 | 15,770 | 1 | 4,054 | AA | AT | 1 |
| comp52603_c0_seq2 | 6,272 | 1 | 2,948 | GG | GA | 1 |
| comp52604_c0_seq12 | 3,167 | 2 | 184 | GG | GA | 1 |
|  |  |  | 190 | GG | GA | 1 |
| comp52608_c0_seq4 | 4,664 | 1 | 3,557 | GG | GA | 1 |
| comp52612_c0_seq2 | 7,856 | 1 | 1,314 | TT | TC | 1 |
| comp52811_c0_seq1 | 1,041 | 1 | 839 | GG | GT | 1 |
| comp53161_c0_seq1 | 1,305 | 1 | 270 | GG | GA | 1 |
| comp53213_c0_seq1 | 1,477 | 1 | 1,404 | CC | CG | 1 |
| comp54189_c0_seq1 | 1,504 | 1 | 1,459 | CC | CT | 1 |
| comp59512_c0_seq1 | 2,506 | 1 | 2,406 | CC | CT | 1 |
| comp14690_c0_seq1 | 542 | 1 | 194 | GC | GG | 2 |
| comp17341_c0_seq1 | 756 | 1 | 523 | CA | CC | 2 |
| comp18290_c0_seq1 | 599 | 1 | 520 | CT | CC | 2 |
| comp18775_c0_seq1 | 2,106 | 1 | 793 | AT | AA | 2 |
| comp20290_c0_seq1 | 1,589 | 2 | 1,045 | TC | TT | 2 |
|  |  |  | 1,136 | CA | CC | 2 |
| comp20344_c0_seq1 | 725 | 1 | 3 | TG | TT | 2 |
| comp20530_c0_seq1 | 1,417 | 1 | 2 | CG | CC | 2 |
| comp20602_c0_seq1 | 991 | 1 | 753 | GA | GG | 2 |
| comp21587_c0_seq1 | 1,653 | 2 | 1,213 | CG | CC | 2 |
|  |  |  | 1,320 | GA | GG | 2 |
| comp21758_c0_seq1 | 964 | 1 | 398 | AC | AA | 2 |
| comp23335_c0_seq1 | 847 | 1 | 714 | GA | GG | 2 |
| comp23666_c0_seq1 | 2,176 | 1 | 555 | GA | GG | 2 |
| comp23800_c0_seq1 | 629 | 1 | 348 | TC | TT | 2 |
| comp25507_c0_seq1 | 417 | 1 | 151 | CT | CC | 2 |
| comp26579_c0_seq2 | 556 | 1 | 2 | TG | TT | 2 |
| comp26830_c0_seq1 | 694 | 1 | 525 | AG | AA | 2 |
| comp29745_c0_seq1 | 1,929 | 1 | 1,098 | AG | AA | 2 |
| comp32821_c0_seq2 | 2,600 | 1 | 1,125 | AG | AA | 2 |
| comp32877_c0_seq1 | 1,787 | 1 | 1,490 | AT | AA | 2 |
| comp32895_c0_seq1 | 4,440 | 1 | 3,922 | GA | GG | 2 |
| comp32915_c0_seq1 | 363 | 3 | 142 | TC | TT | 2 |
|  |  |  | 156 | CG | CC | 2 |
|  |  |  | 158 | TA | TT | 2 |
| comp33157_c0_seq1 | 1,544 | 1 | 1,472 | GC | GG | 2 |
| comp33377_c0_seq1 | 1,211 | 1 | 806 | TC | TT | 2 |
| comp33379_c0_seq1 | 1,681 | 1 | 882 | AG | AA | 2 |
| comp33920_c0_seq1 | 1,248 | 1 | 457 | GT | GG | 2 |
| comp34083_c0_seq1 | 1,921 | 1 | 392 | GT | GG | 2 |
| comp34864_c0_seq1 | 1,497 | 1 | 1,270 | AT | AA | 2 |
| comp35610_c0_seq1 | 1,950 | 2 | 150 | TA | TT | 2 |
|  |  |  | 1,853 | TC | TT | 2 |
| comp39218_c0_seq1 | 1,435 | 1 | 3 | AC | AA | 2 |
| comp39456_c0_seq1 | 619 | 2 | 55 | AC | AA | 2 |
|  |  |  | 302 | AG | AA | 2 |
| comp39557_c0_seq1 | 1,846 | 1 | 1,083 | AC | AA | 2 |
| comp41102_c0_seq1 | 1,427 | 3 | 486 | CT | CC | 2 |
|  |  |  | 1,013 | AG | AA | 2 |
|  |  |  | 1,257 | AG | AA | 2 |
| comp41113_c0_seq1 | 1,749 | 1 | 285 | GA | GG | 2 |
| comp41560_c0_seq1 | 581 | 1 | 176 | CT | CC | 2 |
| comp41615_c0_seq2 | 947 | 2 | 243 | TC | TT | 2 |
|  |  |  | 913 | CT | CC | 2 |
| comp42154_c0_seq1 | 4,481 | 2 | 633 | GA | GG | 2 |
|  |  |  | 3,147 | TC | TT | 2 |
| comp42352_c0_seq1 | 3,051 | 1 | 1,789 | AT | AA | 2 |
| comp42461_c0_seq1 | 403 | 1 | 337 | CT | CC | 2 |
| comp42571_c0_seq1 | 498 | 1 | 102 | TG | TT | 2 |
| comp42892_c1_seq1 | 4,493 | 1 | 340 | TC | TT | 2 |
| comp43346_c0_seq1 | 972 | 1 | 255 | AT | AA | 2 |
| comp43423_c0_seq3 | 910 | 3 | 524 | CT | CC | 2 |
|  |  |  | 625 | CT | CC | 2 |
|  |  |  | 627 | AG | AA | 2 |
| comp43796_c0_seq2 | 1,476 | 1 | 235 | AG | AA | 2 |
| comp44064_c0_seq1 | 1,785 | 1 | 618 | GT | GG | 2 |
| comp44081_c0_seq2 | 184 | 1 | 163 | CT | CC | 2 |
| comp45136_c0_seq1 | 2,058 | 3 | 128 | TC | TT | 2 |
|  |  |  | 383 | TG | TT | 2 |
|  |  |  | 740 | TC | TT | 2 |
| comp45616_c0_seq1 | 596 | 1 | 537 | GT | GG | 2 |
| comp45718_c0_seq1 | 963 | 1 | 663 | TG | TT | 2 |
| comp46233_c0_seq1 | 1,955 | 1 | 394 | AC | AA | 2 |
| comp46651_c0_seq1 | 1,187 | 1 | 1 | GC | GG | 2 |
| comp46713_c0_seq2 | 183 | 1 | 159 | TC | TT | 2 |
| comp46936_c1_seq2 | 2,704 | 1 | 253 | AG | AA | 2 |
| comp47199_c0_seq1 | 1,016 | 1 | 462 | TC | TT | 2 |
| comp47720_c0_seq4 | 1,097 | 1 | 589 | GA | GG | 2 |
| comp47883_c0_seq3 | 725 | 1 | 395 | AT | AA | 2 |
| comp48609_c0_seq3 | 740 | 1 | 219 | CT | CC | 2 |
| comp48615_c0_seq1 | 1,070 | 1 | 905 | AC | AA | 2 |
| comp48642_c0_seq2 | 682 | 1 | 272 | TA | TT | 2 |
| comp49028_c0_seq1 | 2,576 | 1 | 641 | GA | GG | 2 |
| comp49416_c0_seq2 | 1,283 | 1 | 376 | GT | GG | 2 |
| comp49417_c1_seq1 | 1,524 | 2 | 478 | CT | CC | 2 |
|  |  |  | 486 | AG | AA | 2 |
| comp49438_c0_seq1 | 753 | 1 | 551 | CT | CC | 2 |
| comp49559_c0_seq12 | 1,622 | 1 | 1,024 | CG | CC | 2 |
| comp49600_c0_seq2 | 860 | 1 | 755 | CT | CC | 2 |
| comp49946_c0_seq5 | 4,014 | 1 | 3,417 | CT | CC | 2 |
| comp50286_c0_seq1 | 1,012 | 1 | 431 | CT | CC | 2 |
| comp50464_c0_seq1 | 1,809 | 1 | 507 | CT | CC | 2 |
| comp50536_c0_seq4 | 618 | 1 | 275 | TG | TT | 2 |
| comp50566_c0_seq2 | 1,647 | 4 | 651 | AG | AA | 2 |
|  |  |  | 652 | TC | TT | 2 |
|  |  |  | 653 | CT | CC | 2 |
|  |  |  | 669 | TC | TT | 2 |
| comp50607_c0_seq4 | 383 | 2 | 46 | AG | AA | 2 |
|  |  |  | 50 | GT | GG | 2 |
| comp50825_c0_seq2 | 2,989 | 1 | 2,609 | TG | TT | 2 |
| comp50840_c1_seq5 | 2,181 | 1 | 1,915 | GT | GG | 2 |
| comp50845_c0_seq4 | 164 | 1 | 93 | CT | CC | 2 |
| comp50876_c1_seq3 | 1,889 | 2 | 1,562 | TC | TT | 2 |
|  |  |  | 1,596 | GT | GG | 2 |
| comp50997_c1_seq4 | 1,893 | 1 | 1,280 | AG | AA | 2 |
| comp51042_c0_seq2 | 1,988 | 1 | 1,356 | AT | AA | 2 |
| comp51150_c2_seq3 | 656 | 1 | 150 | CT | CC | 2 |
| comp51172_c0_seq1 | 2,276 | 1 | 759 | TC | TT | 2 |
| comp51199_c0_seq1 | 3,311 | 1 | 3,209 | GT | GG | 2 |
| comp51244_c0_seq16 | 2,350 | 2 | 1,264 | TG | TT | 2 |
|  |  |  | 1,270 | AT | AA | 2 |
| comp51479_c3_seq11 | 3,263 | 1 | 3,085 | AT | AA | 2 |
| comp51566_c0_seq1 | 1,187 | 1 | 1,034 | TC | TT | 2 |
| comp51603_c0_seq1 | 831 | 1 | 350 | CA | CC | 2 |
| comp51624_c4_seq1 | 1,678 | 2 | 137 | GA | GG | 2 |
|  |  |  | 568 | TC | TT | 2 |
| comp51630_c4_seq7 | 1,867 | 1 | 199 | GA | GG | 2 |
| comp51680_c1_seq1 | 408 | 3 | 280 | CT | CC | 2 |
|  |  |  | 284 | GA | GG | 2 |
|  |  |  | 351 | GA | GG | 2 |
| comp51770_c0_seq1 | 807 | 1 | 605 | TC | TT | 2 |
| comp51790_c0_seq15 | 2,402 | 2 | 172 | CT | CC | 2 |
|  |  |  | 716 | GT | GG | 2 |
| comp51815_c0_seq19 | 1,270 | 1 | 709 | TC | TT | 2 |
| comp51877_c0_seq1 | 1,311 | 1 | 564 | TC | TT | 2 |
| comp51888_c0_seq6 | 1,360 | 1 | 75 | AC | AA | 2 |
| comp51903_c0_seq4 | 2,162 | 1 | 100 | AG | AA | 2 |
| comp51920_c0_seq3 | 2,792 | 1 | 1,993 | AT | AA | 2 |
| comp51956_c0_seq9 | 1,005 | 1 | 403 | CG | CC | 2 |
| comp51963_c4_seq5 | 2,371 | 3 | 1,467 | CA | CC | 2 |
|  |  |  | 1,534 | AC | AA | 2 |
|  |  |  | 1,564 | AG | AA | 2 |
| comp52014_c0_seq3 | 2,111 | 1 | 1,670 | GT | GG | 2 |
| comp52017_c1_seq10 | 548 | 2 | 428 | CT | CC | 2 |
|  |  |  | 437 | AG | AA | 2 |
| comp52041_c0_seq1 | 2,478 | 5 | 1,624 | TC | TT | 2 |
|  |  |  | 1,627 | TA | TT | 2 |
|  |  |  | 1,690 | CG | CC | 2 |
|  |  |  | 1,751 | AG | AA | 2 |
|  |  |  | 1,753 | CT | CC | 2 |
| comp52065_c0_seq1 | 874 | 1 | 737 | GA | GG | 2 |
| comp52123_c1_seq2 | 1,702 | 5 | 1,379 | TC | TT | 2 |
|  |  |  | 1,456 | TC | TT | 2 |
|  |  |  | 1,491 | AT | AA | 2 |
|  |  |  | 1,493 | AT | AA | 2 |
|  |  |  | 1,494 | AG | AA | 2 |
| comp52156_c0_seq1 | 2,275 | 1 | 661 | TC | TT | 2 |
| comp52282_c3_seq8 | 1,450 | 1 | 748 | GA | GG | 2 |
| comp52353_c0_seq20 | 3,797 | 1 | 3,514 | CT | CC | 2 |
| comp52367_c1_seq2 | 4,730 | 1 | 2,572 | TA | TT | 2 |
| comp52406_c0_seq7 | 700 | 2 | 285 | TC | TT | 2 |
|  |  |  | 571 | TC | TT | 2 |
| comp52481_c0_seq4 | 1,109 | 1 | 474 | TC | TT | 2 |
| comp52506_c0_seq4 | 571 | 1 | 283 | GC | GG | 2 |
| comp52513_c0_seq60 | 786 | 1 | 242 | AG | AA | 2 |
| comp52526_c1_seq7 | 2,638 | 1 | 1,041 | TA | TT | 2 |
| comp52528_c2_seq52 | 1,341 | 1 | 370 | GT | GG | 2 |
| comp52545_c0_seq18 | 2,417 | 2 | 1,054 | AG | AA | 2 |
|  |  |  | 1,055 | GA | GG | 2 |
| comp52582_c0_seq82 | 1,341 | 2 | 492 | TC | TT | 2 |
|  |  |  | 495 | GA | GG | 2 |
| comp52584_c0_seq4 | 3,298 | 1 | 1,213 | GA | GG | 2 |
| comp52603_c0_seq2 | 6,272 | 1 | 2,047 | GT | GG | 2 |
| comp52607_c0_seq3 | 7,710 | 1 | 4,441 | GA | GG | 2 |
| comp52611_c0_seq5 | 5,720 | 1 | 2,122 | AG | AA | 2 |
| comp52612_c0_seq2 | 7,856 | 3 | 4,273 | TA | TT | 2 |
|  |  |  | 7,219 | AG | AA | 2 |
|  |  |  | 7,271 | AT | AA | 2 |
| comp52613_c0_seq4 | 9,696 | 1 | 3,553 | TA | TT | 2 |
| comp52725_c0_seq1 | 766 | 1 | 228 | GC | GG | 2 |
| comp53806_c0_seq1 | 1774 | 2 | 553 | TA | TT | 2 |
|  |  |  | 1233 | CT | CC | 2 |
| comp54533_c1_seq1 | 1443 | 1 | 5 | TA | TT | 2 |
| comp57466_c0_seq1 | 2062 | 1 | 622 | AG | AA | 2 |
| comp58962_c0_seq1 | 2581 | 1 | 2102 | AG | AA | 2 |
| comp59007_c0_seq1 | 2529 | 1 | 279 | GA | GG | 2 |
| comp65310_c0_seq1 | 351 | 1 | 4 | GC | GG | 2 |

^†^Segregation patterns are defined in Table 1.
