# Supplementary material for: Molecular evidence for recent divergence of X- and Y-linked gene pairs in Spinacia oleracea L
Source: PLoS One. 2019 Apr 9;14(4):e0214949. doi: 10.1371/journal.pone.0214949 (PMC6456208; doi:10.1371/journal.pone.0214949)
Supplement: S8 Table — (DOCX) [file pone.0214949.s010.docx]

S8 Table. Expression ratio between alternative alleles at sex-chromosomal loci in male plants.

| Unigene loci ^a^ | Number of loci | Expression ratio | | | *W* | *p* |
| --- | --- | --- | --- | --- | --- | --- |
|  |  | Mean | SD | SE |  |  |
| Sex-chromosomal | 43 | 0.98 | 0.41 | 0.06 | 211.5^c^ | 0.68^c^ |
| Pseudoautosomal | 13 | 0.98 | 0.23 | 0.06 | 60^d^ | 0.95^d^ |
| Y/X linked | 9 | 0.99^b^ | 0.12^b^ | 0.04^b^ |  |  |

SD, standard deviation; SE, standard error.

^a^ Unigene loci with multiple sex-linked SNPs (n > 1) heterozygous in all male plants used for the RNA-seq analysis were evaluated.

^b^ Ratios of Y/X expression are indicated.

^c^ Wilcoxon exact rank sum test for the comparison between the sex-chromosomal and Y/X-linked loci.

^d^ Wilcoxon exact rank sum test for the comparison between the pseudoautosomal and Y/X-linked loci.
